# Creating a shared vision for change in a complex system

**Published:** 2025-12-02

**Authors:** Anna McKeon, Samit Sakib Gore

**Affiliations:** 1 Managing Director, Dialogue: Thinks Insight & Strategy, London, UK.; 2 Director of Operations and Innovation: Vision Friend Sakib Gore, Badlapur, India.

**A shared vision builds momentum by making it easier to mobilise support and influence decision makers**.

**Figure:**
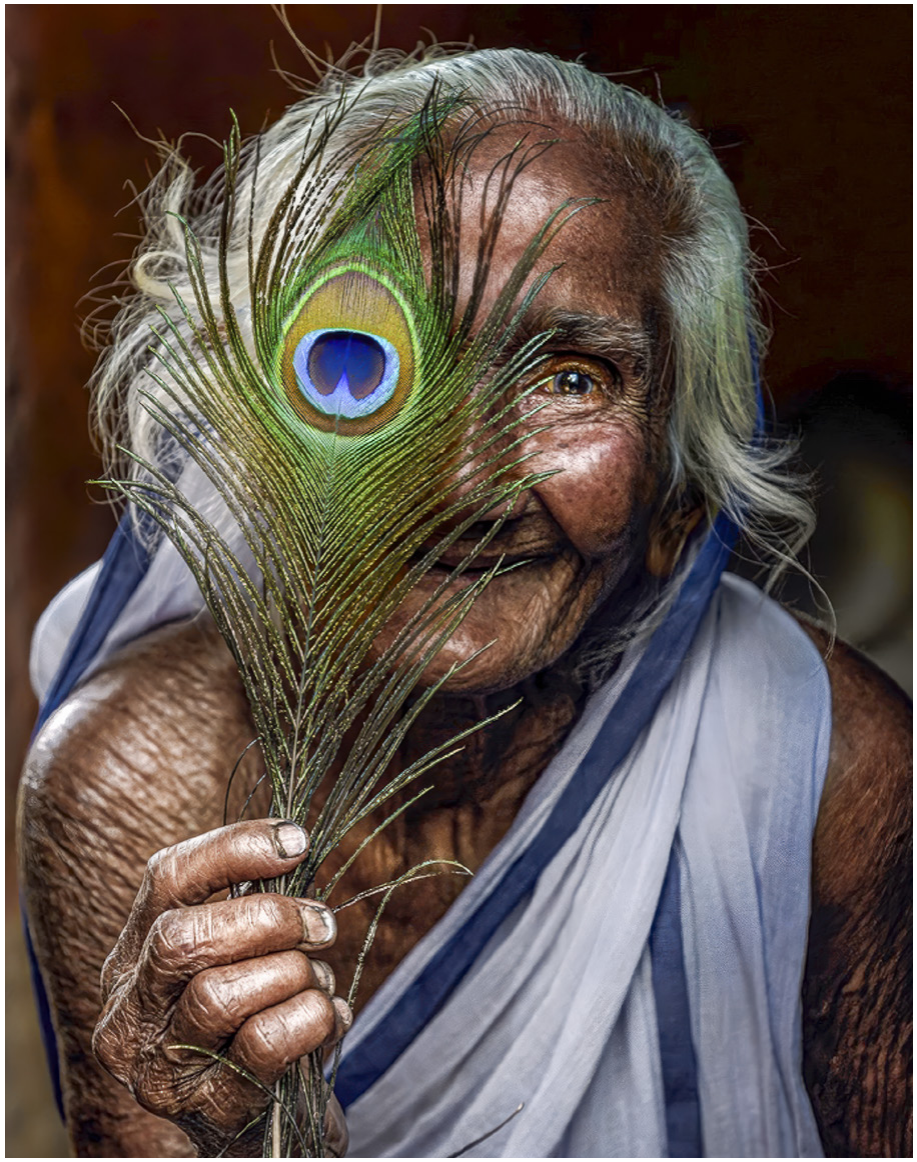
In complex systems, perspective is everything. Leadership is the art of seeing it all and finding a shared lens.

© SHIBASISH SAHA, COURTESY OF IAPB CC BY-NC-SA 4.0

In the first article in this series (bit.ly/3WB06Ru), we explored the need for a new kind of leadership in eye health and introduced systems thinking as a foundational skill. In the second article (bit.ly/3Linvms), we looked at the first core leadership skill: systems thinking (what makes systems complex, how cause and effect aren't always obvious, and how change emerges through relationships). In this article, we focus on the second core leadership skill: strategic vision.

## Why a shared vision matters

In complex and unpredictable environments, it’s easy to get stuck reacting to problems as they arise. Without a shared sense of direction, such reactive efforts remain fragmented and short-term in nature. One of the most powerful things we can do as leaders is to help shape a shared purpose that enables diverse people to work together in the same direction.

A compelling vision acts as a compass, helping people make sense of uncertainty, understand the contribution their daily work makes, and align around what matters most. It helps them see how their roles fit into a bigger movement, builds momentum, and enables collective action across diverse stakeholders. It provides a common foundation for policy asks, communications, and campaigns. A shared vision builds momentum by connecting local actions to broader change, making it easier to mobilise support and influence decision-makers. Most importantly, it gives people a reason to believe that their efforts are part of something bigger.

## Understanding root causes

Before defining a vision, effective leaders take time to explore the deeper forces shaping the current situation. This means considering what the root causes of problems might be, and what the forces for and against change are. Tools, like multiple cause diagrams and force-field analyses, can help us to identify patterns and power dynamics that aren't otherwise easy to see. This process is often best done collectively, involving people from across the system, to gain multiple perspectives and build shared understanding.

It is helpful to do this kind of exploration before developing a shared vision, because having a shared understanding of the problem is a key part of generating real insight and collective buy-in for the path forward. Through this process, we stop reacting and begin rethinking.

This idea builds directly on our second article’s emphasis on systems mapping, where we zoom out to understand relationships, power, and the wider context. Without this, even the most well-intentioned vision can feel disconnected from reality.

**Previous articles in this series**

1. Leadership for 2030 In Sight: defining the skills needed to drive change bit.ly/3WB06Ru
2. Systems leadership for sustainable change bit.ly/3Linvms

## Defining a bold, actionable vision

A vision is not a slogan, or an aspirational statement. It is a vivid description of a future you are actively working to create. IAPB’s sector strategy, 2030 In Sight, has a strong vision statement: “A world in which everyone has access to the best possible standard of eye health; where no one is needlessly visually impaired; and where those with irreparable vision loss achieve their full potential.”

Strong vision statements clearly identify who will experience the change, when the change is hoped to occur, and how it connects to other global frameworks. They are ambitious yet grounded, and offer a roadmap for others to align with.

Strong vision statements should:

- Clearly define **who** will experience the change
- Indicate **by when** we hope the change will happen
- Describe a realistic **future**
- Be **linked** to other initiatives.

For example, in the IAPB sector strategy, 2030 In Sight, **who** will experience the change is everyone; **by when** is 2030; and the **future** described is one where no-one is “needlessly” visually impaired – a more realistic goal than one in which no-one at all is visually impaired. Finally, the language used **links** to that used in the World Health Organization's statements on universal health coverage.

Creating such a vision means balancing big-picture ambition with feasibility. Leaders must be able to navigate competing priorities, align stakeholders, build coalitions, and identify and create strategic milestones along the way that keep the vision alive.

## Communicating for mobilisation

Even the most compelling vision has little impact if it remains in a document or PowerPoint slide. These don't inspire, but stories do. To mobilise people, we must build tools that turn statistics into lived experience. Leaders must communicate it in ways that inspire others to act.

This means:

- Using simple, relatable language
- Connecting the vision to people’s values and lived experiences
- Sharing stories that bring the vision to life
- Listening actively to build ownership and alignment.

Importantly, vision-building is not a one-time event. It’s a continual process of engaging others, adapting to new realities, and reinforcing a shared purpose. This is because true mobilisation happens when people feel they are part of something meaningful and achievable.

## Case study: Vision Friend Sakib Gore

‘Vision Friend Sakib Gore’ (Vision Friend) is a grassroots initiative driven by a powerful idea: that **clear sight is a basic right, not a privilege**. This clarity of purpose – that vision should be for all – became a powerful rallying point. It resonated across communities and cultures, and inspired thousands to get involved.

Founded by Sakib Gore, a former truck labourer from a farming village in India, Vision Friend has evolved into a movement, not because of strategy or funding, but because the vision was bold, inclusive, and personal. Since 1992, the organisation has reached over 1,700 villages across India, conducted over 2.7 million free eye tests, distributed 1.6 million free spectacles, and facilitated 63,000 free cataract surgeries, all without relying on external funding.

Vision Friend’s success in mobilising people and resources is closely tied to how it communicates. Rather than simply raising awareness, or sharing data points, the organisation uses human stories and experiences to create empathy and drive action.

These aren't one-way messages, either: Vision Friend invites people, whether policymakers, funders, or community members, to attend multisensory events where they can experience how vision loss feels and what is possible when sight is restored. Events are held in villages, camp locations, factories, educational institutions, and cities.

Some of the tools Vision Friend uses at these events include:

- **Cataract simulation glasses.** These help decision-makers and the public understand the disorientation, dependency, and danger of preventable blindness, transforming a clinical concept into an emotional, lived experience.
- **Documentary storytelling.** *In Pursuit of Light*, a documentary co-created with volunteers, traces Vision Friend’s journey from one labourer’s mission to a global movement. By explaining systems change through a personal story, the film invites people to relate and to act.
- **Immersive experiences:** Augmented reality, virtual reality, and anatomical eye models offer a visceral way for funders and policymakers to engage with the issue, making it harder to ignore and easier to champion.

**Figure:**
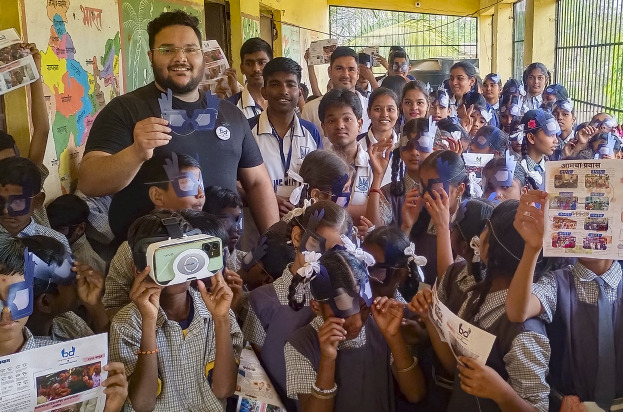
During an immersive eye health awareness campaign at a school in Thane District, Vision Friend Sakib Gore used augmented reality devices, cataract simulation glasses, and anatomical eye models to help children understand how vision works. They also screened the film *In Pursuit of Light* and distributed printed information about refractive disorders and treatable causes of blindness. INDIA

© AAMAN SHAIKH, VISION FRIEND SAKIB GORE CC BY-NC-SA 4.0

These approaches have helped shift public and political perceptions from passive awareness to active engagement. People don't just learn about eye health; they see themselves in the story and choose to become part of the solution.

At every level, Vision Friend’s messaging reinforces the shared vision: a vision that communities can see themselves in, and one that others can adopt, adapt, and carry forward.

## What does this mean for eye health professionals?

This story reminds us that sustainable change doesn't start with funding or infrastructure, it starts with a clear, shared purpose that people can believe in and act on. For eye health professionals, especially in low- and middle-income settings, that insight translates into some practical steps:

- **Start with the ‘why’.** Make the purpose of your work visible and relatable. Is your message about dignity? Opportunity? Inclusion? People mobilise around values, not just services.
- **Co-create the vision.** Ask people what a future with accessible eye health looks like to them, and let their ideas shape your work.
- **Use simple tools to spark empathy.** These can include cataract simulation glasses. Think creatively about how to help others understand the problem. Visual, hands-on experiences can do more to mobilise support than any report.
- **Use stories as well as statistics.** As with the volunteer-made documentary, real stories of transformation help people see what’s possible – and see their role in it. Make space for case studies and community voices in your communications.
- **Build for longevity, not dependency.** Invest in leadership and ownership within communities; for example, local volunteers, peer educators, or youth groups who can carry the vision forward without you.

Ultimately, this is about mobilising people: not around a service, but around a purpose – one they feel they belong to and can contribute to. That is how you build movements that last.

